# Music-Based Therapeutic Interventions for People with Dementia: A Mini-Review

**DOI:** 10.3390/medicines5040109

**Published:** 2018-10-08

**Authors:** Annemieke Vink, Suzanne Hanser

**Affiliations:** 1 Music Therapy Department, ArtEZ University of the Arts, Academy of Music, 7511 PN Enschede, The Netherlands; 2 Music Therapy Department, Berklee College of Music, Boston, MA 02215, USA

**Keywords:** music therapy, music-based interventions, dementia, systematic reviews, clinical practice, mini-review

## Abstract

The growing population of people with dementia worldwide calls attention to the burdens associated with age-related decline that affects physiology, psychological and cognitive status, and social/emotional wellbeing. The current standards in geriatric care recommend non-pharmacological approaches to these challenges, including safe approaches to managing pain and stress, enhancing symptom relief, and fostering independent lifestyles with the highest quality of life possible. The purpose of this article is to provide definitions of music-based interventions, music therapy applications and clinician qualifications, as well as an umbrella mini-review of meta-analyses regarding music-based interventions for individuals with dementia. Our findings indicate that most descriptions of music therapy protocols in the research lack sufficient detail to enable researchers to compare and replicate studies, and clinicians to apply techniques. Definitions of music therapy and music-based interventions are inconsistent, and practitioners vary in their professional training and preparation for implementing music-based clinical strategies. We recommend that future researchers provide thorough descriptions of music therapy and music-based interventions in their protocols.

## 1. Introduction

### 1.1. Music and Dementia

With the recognition that music is a preserved skill in many individuals who have dementia [1,2], most residential settings for elder care have made a variety of music activities and services part of their regular programming. These encompass a range of opportunities, including entertainment by local musicians and performers, background music piped into public areas, use of individual music listening devices, and specific clinical interventions by trained music therapists, to name a few. Since music is so easily accessible, obtainable in multiple cultures and languages, unregulated, and capable of being simply applied (as in the case of music listening), almost anyone can develop a “music protocol” and test it in a clinical trial. The aim of this paper was to examine definitions of music-based interventions, music therapy applications and clinician qualifications, as they appear in the literature. With the intention of identifying potentially effective music-based interventions, the investigators conducted a cursory analysis of research studies, as well as an umbrella mini-review of the interventions that are published in studies from meta-analyses.

### 1.2. What Is Music Therapy

In recent years, the professional practice of music therapy has gained attention as a treatment approach for dementia care. In the USA, music therapy is defined by the American Music Therapy Association (AMTA) as “the clinical and evidence-based use of music interventions within a therapeutic relationship to accomplish individualized goals by a credentialed professional who has completed an approved music therapy program” [3]. This definition of music therapy and the accompanying AMTA Standards of Practice identify an individualized treatment process, including referral, building a therapeutic relationship, assessment, observation, targeting individualized goals and objectives, treatment planning, protocol selection and implementation, termination, and evaluation [4]. This process is conducted by a qualified music therapist who masters these essential steps of offering music interventions in therapeutic treatment, and upon completion of AMTA requirements, can apply for national board-certification [5]. Music therapy operates on a strength-based, data-driven model, i.e., skills and abilities are built based on an assessment of the individual’s needs, clinical observations, and preferences/responsiveness to different music-based interventions. The AMTA Scope of Practice document lists certain interventions that may be indicated, but this limited inventory only hints at the multiplicity of potential protocols: “music improvisation, receptive music listening, songwriting, lyric discussion, music and imagery, singing, music performance, learning through music, music combined with other arts, music-assisted relaxation, music-based patient education, electronic music technology, adapted music intervention, and movement to music” [6]. Obviously, protocols are tailored to individual needs; yet adherence to this clinical methodology runs contrary to the development of standardized protocols for implementation in clinical trials.

The definition of music therapy by the World Federation of Music Therapy is described as ”the professional use of music and its elements as an intervention in medical, educational, and everyday environments with individuals, groups, families, or communities who seek to optimize their quality of life and improve their physical, social, communicative, emotional, intellectual, and spiritual health and wellbeing. Research, practice, education, and clinical training in music therapy are based on professional standards according to cultural, social, and political contexts” [7]. This definition references spiritual health, a variable that is decidedly challenging to measure, and emphasizes the importance of culturally-informed practice. Thus, protocols developed by music therapists who present their own distinctive cultural backgrounds and global identities can reflect vastly different approaches and practices around the world. This global definition does not include the qualifications of a music therapist, perhaps in recognition of the disparate training, standards, and practice models in individual countries.

### 1.3. The Advantages of Music Therapy

The benefits of utilizing music therapy strategies are multi-faceted. First, music therapy is one of the few therapeutic approaches that can be offered via intensive individual treatment, as well as for a group of individuals who demonstrate varying levels of functioning. It is practiced in community and home settings, as well as in long-term care. Many therapeutic approaches for the problems associated with dementia rely on speech and language. However, when verbal communication is not possible, music therapy offers musical instruments and improvisation for the expression of emotions, and music listening to evoke changes in mood and responsiveness. Throughout neurodegenerative loss, such as in cases of Alzheimer’s or Parkinson’s diseases, music therapy may accompany the individual from early diagnosis into the final stages of these and other similar conditions [8]. The therapeutic relationship and the ensuing reciprocal interaction both contribute to the ability of music to influence behavior, but of course, these factors add sources of variability to an experimental design. Finally, music therapy is non-invasive, safe, and largely without side effects.

### 1.4. Other Music-Based Interventions

In a review of music interventions in neurologic rehabilitation, Sihvonen et al. [9] categorized music-based interventions as any protocols using music, whereas music medicine is any music-based intervention delivered by a healthcare professional. Music-supported therapy included interventions for neurorehabilitation where musical instruments assist in the movement of the extremities. Rhythmic auditory stimulation and melodic intonation therapy are two well-defined research protocols for movement rehabilitation and non-fluent aphasia, respectively. These are unique in providing specific practice details, facilitating standardized implementation and replication. Given the wide diversity in characterizing music-based interventions, we sought to more carefully identify those interventions that met the criteria for inclusion in systematic reviews and meta-analyses.

## 2. Methods

In order to identify examples of music therapy protocols and music-based interventions being researched for people with dementia, we engaged first in a cursory examination of the individual studies included in systematic reviews (Phase I). We also attempted to analyze current trends and developments in types of music interventions. Subsequently, we conducted an umbrella mini-review of the music-based interventions that had been studied in meta-analyses (Phase II). 

In Phase I, we examined individual studies of music-based interventions for people with dementia that were included in review articles and systematic reviews published between January 2015 and August 2018. Searches were carried out using PubMed/Medline, Google Scholar, EBSCOhost Complete, Embase, and Cinahl. Based on the abstract and article content, we retrieved 11 eligible systematic reviews published since 2015. These included: Elliott and Gardner [10]; Fakhoury et al. [11]; Fusar-Poli et al. [12]; Gomez-Romero et al. [13]; Ing-Randolph, Phillips and Williams [14]; Pedersen et al. [15]; Petrovsky, Cacchione and George [16]; Tsoi et al. [17]; van der Steen et al. [18,19]; and Zhang et al. [20]. 

In Phase II, we conducted an umbrella mini-review of the six systematic reviews that included meta-analyses. This umbrella review was conducted by examining the specific interventions applied in individual studies that met the criteria for inclusion in the most recent systematic reviews. These six were conducted by Fusar-Poli et al. [12], Pedersen et al. [15], Tsoi et al. [17], van der Steen et al. [18,19], and Zhang et al. [20]. The results of the umbrella review are presented in narrative form.

## 3. Results

### 3.1. Phase I: Examples of Interventions

In the cursory examination of the 11 systematic reviews on the topic, the number of studies examining the effects of music therapy for people with dementia increased over time. In a Cochrane review in 2004 on the effects of music therapy in dementia care [21], only six studies were included, whereas in the latest update in 2018, 22 studies met the criteria for review [19]. In the 2004 version, however, only music therapy studies were included. The 2018 review included articles that reported music interventions with a therapeutic intent including, but not limited to, interventions provided by qualified music therapists. The reported outcomes included changes in cognition, agitation, disruptive behavior, emotional well-being, quality of life, and social behaviors.

Active engagement with music was observed to have a strong influence on individuals with varying degrees of dementia. In one study, singing well-known songs triggered long-term memories and feelings associated with the people, places and conditions that were present when these songs were learned. With individuals whose basic orientation to time, place and identity was failing, singing familiar music was shown to evoke positive changes in mood and social behavior [22]. Singing was central to an effective intervention for relearning speech by those individuals who demonstrated aphasia [23]. At the other extreme, passive music listening without intervention by a trained therapist also resulted in significant effects, e.g., anxiety reduction due to listening to preferred music in a long-term care facility in Taiwan [24]. In Japan, listening to preferred songs sung by a music therapist with some verbal intervention resulted in the release of 17 β-estradiol and testosterone, hormones that have been implicated in the exacerbation of Alzheimer’s disease [25].

Raglio and Oasi [26] characterized music therapy interventions as relational music therapy and rehabilitative music therapy. The former realizes the importance of the therapeutic relationship, as supported by psychological and neuroscientific theory; the latter emphasizes specific aims and techniques (mostly active) to address them. These authors referred to music listening approaches as individualized music listening vs. music medicine aimed towards alleviating selected symptoms and conditions. 

While music therapy protocol descriptions in published randomized controlled trials (RCTs) often lack the specificity and individualization required by practice standards, other music-based interventions may include broadly-defined uses of music. Tested protocols include many commercial programs that have become popular after press releases and YouTube videos that demonstrated people “coming alive” with music, such as in the “Music and Memory” program [27]. Training is available for “Music and Memory” facilitators, where “music practitioners” can be certified to provide bedside music for healing and transition [28]. Further, iPhone applications, e.g., “Spark Memories Radio” [29] and “SingFit” [30] are marketed as ways for caregivers to offer music to their family members or residents with dementia. Unfortunately, without rigorous evidence and guidelines on how to apply these interventions with people with dementia, it is possible for consumers to be misled by testimonials and unresearched claims of effectiveness on some websites and in popular advertisements. 

The published literature suffers from lack of clarity in types of practitioners as well as interventions. Choi et al. [31] describe group music interventions for dementia in the title of their article, while the intervention is being offered by music therapists. Other titles refer to “the effect of music therapy” while the interventions include music listening or group singing without the presence of a qualified music therapist [32]. There is limited information on the differential effects of music intervention offered by a nurse, music therapist, or other professionals. Similar issues arise in labeling music-based interventions outside of dementia care, notably in medical settings [33].

### 3.2. Music in Clinical Settings

The vast majority of research is performed in long-term care facilities where residents are available for clinical interventions. However, community-based strategies and family caregiver-conducted programs hold potential for delaying placement in a skilled nursing facility or residence, a significant outcome affecting costs and quality of life for people with dementia. Experimental control may be compromised when studying individuals who reside in the community or at home, due to the many uncontrolled variables in these settings. This may account for the limited research in this area. Music-based interventions for dementia are designed primarily for the many psychosocial and behavioral symptoms that plague those who exhibit the distress and confusion that defines dementia.

### 3.3. Phase II: Umbrella Mini-Review of Meta-Analyses

The umbrella mini-review more thoroughly examined the types of music-based interventions for dementia included in the six meta-analyses. Each of the studies reviewed by Fusar-Poli et al. [12] tested a different music protocol and its effects on cognition. These included: exercises to stimulate attention and memory (STAM-Dem); playing, listening and singing with instrumental accompaniment; “U Sequence” method involving listening to specific sequences of musical selections; listening to preferred music; nonverbal music therapy to promote intersubjective communication; and discussing music that elicited emotions, thoughts and memories. Pedersen et al. [15] focused on interventions for agitation, and included: favorite music playing during baths, music streaming in the background, individualized music or music therapy sessions for various periods of time, 10-min exposure to music; and listening to prescribed music. Tsoi et al. [17] examined the behavioral and psychological symptoms of dementia in response to music therapy interventions that were active or receptive approaches but did not further differentiate between interventions. The two Cochrane reviews by van der Steen et al. [18,19] applied the most exclusive criteria for interventions in their meta-analyses on dementia in general. They only included therapeutic music-based interventions, defined as “therapy provided by a qualified music therapist, or interventions based on a therapeutic relationship and meeting at least two of the following criteria/indicators: (1) therapeutic objective which may include communication, relationships, learning, expression, mobilization, and other relevant therapeutic objectives; (2) music matches individual preferences; (3) active participation of the people with dementia using musical instruments or singing; and (4) participants had a clinical indication for the intervention or were referred for the intervention by a clinician” [33]. In their earlier review, van der Steen et al. [18] were unable to determine the qualifications of those who delivered the music in 4 out of 22 studies, and cited difficulties in determining the education or experience of the clinician or person who administered the protocol. Zhang et al. [20] did not differentiate between interventions that were conducted by qualified music therapists and other music techniques but reported the use of interactive vs. passive music approaches.

### 3.4. Summary/Interpretation of Findings

Most of the systematic reviews concluded that music therapy or music-based interventions can be beneficial for the care of people with dementia. Ideally, clinicians wish to apply the recommendations of the reviews and their guidelines into their practices. However, this is complicated. There are many differences between the studies included in these reviews with regard to design, measurements, outcomes, and conclusions. A welcome contribution in recent Cochrane reviews is that future articles should specifically address agreements and disagreements between individual studies in their analyses. Van der Steen et al. [19] provide a detailed overview in their recent Cochrane review in 2018. 

The specific inclusion criteria for each review accounts for many of the differences between the conclusions that are reached, e.g., the types of research designs that are accepted for inclusion. When reviewing the reviews that included meta-analysis techniques summarizing the effect of music based interventions, van der Steen et al. [18,19] found little to no effect for music-based interventions in reducing agitation, based on randomized studies only, whereas Tsoi at al. [17] and Zhang et al. [20] found music therapy to be moderately effective, based on the inclusion of non-randomized studies, as well as randomized studies. Search terms in specific languages further influence the inclusion of studies, e.g., van der Steen et al. [18,19] reviewed articles in English, French, German, and Dutch, which is far more inclusive than in articles by non-Europeans. Most importantly, the inclusion criteria and definitions for music therapy and music-based interventions vary greatly, making transfer to clinical practice even more challenging. In addition, specific interventions being offered are generally described with little detail. 

This mini-review also indicates diversity in the potential outcomes of musical interventions, and calls attention to the importance of cultural considerations in the delivery of music interventions and music therapy. Given the significant psychosocial and behavioral needs of individuals who display dementia, music-based interventions have largely been targeted to promoting changes in disruptive behavior and agitation, as well as enhancing cognition and quality of life in general, as opposed to medical outcomes.

Based on the latest published Cochrane review [19], the main conclusion is that providing people with dementia who are in institutional care with at least five sessions of a music-based therapeutic intervention probably reduces depressive symptoms and improves overall behavioral problems at the end of treatment. It may also improve emotional well-being and quality of life and reduce anxiety but may have little to no effect on agitation, aggression or cognition.

There are multiple issues that result in difficulties in interpreting and applying these findings and those of other meta-analyses. RCTs require standardized protocols to facilitate the replication and control of external variables; however, these may not take into account individual needs—the basis for music therapy protocol development—nor might they be clinically or culturally relevant to the people being treated. Systematic reviews and their conclusions are based on the quality and contents of individual RCTs.

## 4. Discussion

The number of studies and reviews in the context of music therapy for people with dementia has been growing rapidly over the past several decades. Unfortunately, individual studies and systematic reviews on the effects of music and music therapy provide limited descriptions of music-based interventions, particularly regarding the qualifications of the practitioner and details regarding the use of music. 

It is recommended that future research states the qualifications of the practitioners, reporting their level of professional education and experience with dementia. Greater detail should be provided for the precise interventions being offered. Often, sessions are described in objective terms, e.g., the duration of the session, group size, instruments, and general repertoire; however, the therapeutic process over the course of treatment, specifically in relation to musical processes, fails to be addressed in most research articles. Developing standardized protocols will facilitate replication for future research agendas. Protocols should be based on scientific knowledge regarding underlying mechanisms, as well as the valuable work and best practices of music therapists and other clinicians over the last 50 years. Researchers should be referred to guidelines for reporting research on music-based interventions [34]. Currently, most studies are conducted within a nursing home facility. For future studies, it would be advisable to investigate the effect of music-based interventions in the home situation, as many people with dementia live at home. More detailed intervention descriptions will better inform clinicians and facilitate the future replication of protocols. Only then will reviewers be able to more clearly describe the specific effects of different interventions.
